# Genotyping faecal samples of Bengal tiger *Panthera tigris tigris *for population estimation: A pilot study

**DOI:** 10.1186/1471-2156-7-48

**Published:** 2006-10-17

**Authors:** Jyotsna Bhagavatula, Lalji Singh

**Affiliations:** 1 Centre for Cellular and Molecular Biology, Uppal Road, Hyderabad 500 007, India

## Abstract

**Background:**

Bengal tiger *Panthera tigris tigris *the National Animal of India, is an endangered species. Estimating populations for such species is the main objective for designing conservation measures and for evaluating those that are already in place. Due to the tiger's cryptic and secretive behaviour, it is not possible to enumerate and monitor its populations through direct observations; instead indirect methods have always been used for studying tigers in the wild. DNA methods based on non-invasive sampling have not been attempted so far for tiger population studies in India. We describe here a pilot study using DNA extracted from faecal samples of tigers for the purpose of population estimation.

**Results:**

In this study, PCR primers were developed based on tiger-specific variations in the mitochondrial cytochrome b for reliably identifying tiger faecal samples from those of sympatric carnivores. Microsatellite markers were developed for the identification of individual tigers with a sibling Probability of Identity of 0.005 that can distinguish even closely related individuals with 99.9% certainty. The effectiveness of using field-collected tiger faecal samples for DNA analysis was evaluated by sampling, identification and subsequently genotyping samples from two protected areas in southern India.

**Conclusion:**

Our results demonstrate the feasibility of using tiger faecal matter as a potential source of DNA for population estimation of tigers in protected areas in India in addition to the methods currently in use.

## Background

The Bengal tiger or *Panthera tigris tigris*, the National Animal of India, is an endangered animal. Though tiger populations were high at the turn of the last century, their numbers have reduced drastically due to hunting/poaching as well as human activities that have resulted in habitat fragmentation and prey depletion [1]. Reliable estimates of tiger populations are essential for designing conservation planning protocols as well as assessing the management plans already in place.

Estimates of tiger numbers, though the figures are controversial, were supposed to be around 4500 in May 1998, spread out all over the Indian-Subcontinent in protected areas [2]. Controversies about tiger numbers have arisen due to the methodology being followed for population estimation. Tigers are territorial, elusive, cryptic and nocturnal animals. The territory of a male is about 25–30 sq kms and it may overlap the territory of several females, which generally have smaller territories of around 10–15 sq kms [3]. Due to their elusive nature, it is not possible to carry out a direct enumeration of tigers. Therefore, indirect methods are the only means of enumerating tigers.

The most commonly used method, 'the pugmark method', has been in use for more than two decades now and attempts at ascertaining the total count of tigers in a protected area. It is based on the assumption that tiger pawprints are unique to individuals [4] and that tracks can be found for all the tigers and recorded at the same time. Annually, the tigers in protected areas in India are 'censused' by Forest Officials by this method. However, several drawbacks of this method have been pointed out and its veracity questioned by tiger researchers. (See [3] for a critique of this method). The other method is the camera-trap technique, which is based on the assumption that stripe patterns on tigers are individual-specific [5]. Camera-trapping techniques have been used for identifying individual tigers [6]. This method has been used for estimating tiger abundance and density in the conceptual framework of the mark-recapture statistics and has been employed in providing estimates of tiger populations in many protected areas in India and elsewhere [7-9]. However, this method works best only in areas where tigers are in high density; it can sample tigers only in a few predetermined locations where camera traps are set, and cannot be used in difficult terrains.

Non-invasive methods of collection of biological samples, such as faecal samples or hair samples, have been successfully employed for population estimation of animals like Brush-tailed rock-wallaby [10], Coyotes [11], Forest elephants [12], European Badgers [13], wolves [14] and grizzly bears [15] with genetic profiles generated using multilocus microsatellite loci unique to individual animals. However, the use of faecal samples as a source of DNA for genotyping has not been attempted for estimating wild tiger populations.

Tigers deposit scats or leave scent marking or scrapes on the soil to demarcate their territories [5]. Therefore, faecal matter of tigers would be the most easily obtainable source of DNA for non-invasive studies of tigers. Taberlet and Luikart [16] have recommended that any study requiring a non-invasive genetic method should be preceded by a pilot study to assess the Probability of Identity, P(ID), as well as the feasibility and reliability of the method before embarking on a large scale study. In addition, the pilot study should demonstrate the error rates and the difficulties like allelic dropouts and false amplifications that can be encountered during the process of genotyping DNA from non-invasive sources [16,17].

Quantity and quality of template DNA is the limiting factor from non-invasive sources [18]. We, therefore compared sample preservation and DNA extraction methods for ascertaining the method best suited for Indian field conditions.

The Probability of Identity, P(ID), is the power of the molecular markers to resolve between different individuals drawn at random from a population [16,19]. Microsatellite markers are the markers of choice in all studies where individual identification is attempted using the non-invasive method of obtaining DNA [20-23]. However, if in a population there are closely related individuals genetic profiles may look similar even in different individuals, especially if less number of genetic markers are used. This is called the 'shadow effect' [24]. Therefore, it is important to screen for markers with a low enough P(ID) in order to resolve siblings and other close relatives in a population. Microsatellite markers with a low probability of P(ID) for reliable individual identification were developed and screened.

Our pilot study was carried out on faecal samples collected from the field to check whether it was possible to carry out faecal sample genotyping in actual field conditions. Identification of the error rate on faecal samples collected from field was evaluated in order to demonstrate the use of DNA extracted from such samples for large-scale genetic studies of tiger using faecal DNA. For this purpose, tiger faecal samples were collected randomly with the help of trackers of the Forest Department from Mudumalai and Biligiri Rangan Temple (BRT) Wildlife Sanctuaries. Samples collected thus can reveal the minimum number of tigers living in the protected area of study at the time of collection. In addition to this, we have also standardized a PCR-based species and sex identification method.

Therefore, the main aim of our pilot study was to optimize protocols to obtain genetic profiles for individually identifying tigers from faecal samples and to demonstrate whether DNA from such a non-invasive source can be used for estimating tiger populations in the wild in India.

## Results

### Faecal sample preservation and DNA extraction

There was no significant difference in DNA extraction and subsequent PCR amplifications between the two sample preservation trials, namely storage desiccant silica and 90% ethanol (p > 0.05, two tailed t-test).

38% extracts from the Chelex-100 method of DNA extraction from faecal samples [25]; 75% from the Digest Buffer/Phenol Chloroform method [26,27]; 25% from the Lysis buffer/column purification method [28]; 88% extracts from Guanidinium thiocyanate-silica method [26]; and 100% extracts from Qiagen Stool DNA extraction kit could be PCR amplified with mitochondrial cytochrome b primers that target a 146 bp fragment [29].

Because less than 50% of the PCRs were positive with the Chelex-100 and Lysis buffer/column purification methods, they were not analyzed further. Extracts from the Digest Buffer/Phenol Chloroform, Guanidinium thiocyanate-silica, and Qiagen Stool DNA extraction kit methods were amplified with five microsatellite primers, namely E21, E7, D10, D15 and Fca304. There was no significant difference in PCR amplifications of microsatellite markers among these three extraction methods. (q > 0.05, Nemenyi's test).

### Molecular identification of faecal samples as tiger in origin

**Figure 1:**
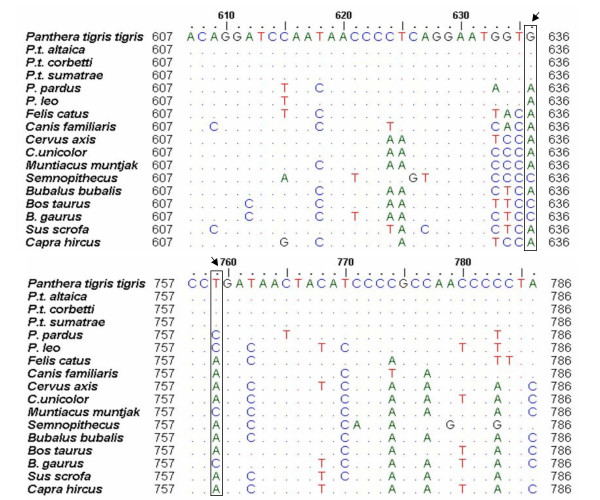
**Sequence alignment of some prey species of tigers, the extant sub-species of tigers and some carnivore species**. Arrows indicate the tiger-specific variations at position 636 and 759 of the tiger mitochondrial cytochrome b gene that were used for designing primers for tiger-specific diagnostic PCR assay.

**Figure 2:**
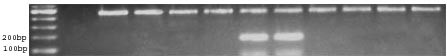
**A representative gel pattern showing the specific amplification of only tiger DNA with the tiger-specific cytochrome b primers (TIF/TIR)**. Lane 1: 100 base pair ladder (New England Biolabs); Lane 2: negative control; Lane 3:*Panthera pardus*; Lane 4:*Cuon alpinus*; Lane 5: *Neofelis nebulosa*; Lane 6:*Cervus unicolour*; Lane 7: *Panthera tigris tigris*; Lane 8: *P.t. altaica*; Lane 9: *Bos gaurus*; Lane 10: *Cervus axis*; Lane 11: *Sus scrofa; Lane *12: *Cervus axis*. PCR amplification with universal 'mcb' primers [48] of all animals tested rules out the possibility of false negatives in PCR with the tiger-specific primer pair (TIF/TIR). Amplification with primers TIF/TIR and mcb primers was carried out in separate reaction though the PCR products were loaded onto a gel together.

Primers TIF/TIR were designed based on tiger-specific variations in the tiger mitochondrial cytochrome b gene. Two unique tiger-specific bases were detected namely a guanine at positions 636 and a thymine at position 759 (Figure 1). Primers were designed with these bases at the 3'-ends which amplify a 162 bp fragment in an *in silico *PCR. Validation of these primers on a panel of DNA samples of different animals shows that they amplify only tiger DNA and not of other species tested (Figure 2).

**Figure 3:**
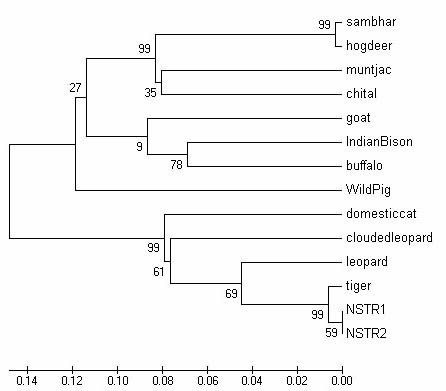
**Neighbour-Joining tree of amplicons of two faecal samples from Nagarjunasagar Srisailam Tiger Reserve (NSTR1 and 2)**. Sequences of amplicons of both samples, which were positive in the tiger-specific diagnostic assay, cluster with GenBank tiger sequence proving further that the samples are tiger in origin.

Two out of eight carnivore scat samples collected from Nagarjunasagar-Srisailam Tiger Reserve (NSTR) were positive for PCR with TIF/TIR. Sequences of these amplicons clustered with *Panthera tigris *in an NJ tree constructed with MEGA, showing further that these samples are tiger in origin (Figure 3).

### Polymorphic microsatellite markers and Probability of Identity

A total of ten microsatellite markers screened were polymorphic (Table 1). Mean Polymorphism Information Content (PIC) was 0.68. Average number of alleles per locus was 6.4. Except loci Fca139 and D15 no linkage disequilibrium was detected for the other possible combination of loci after Bonferroni correction. Significant deviation from Hardy-Weinberg equilibrium was detected for loci D15, C34, 6HDZ003 and Fca139.

**Table 1:** Polymorphic microsatellite markers that were screened on a panel of captive tigers (n = 21)

Locus	Primer Sequence (5'-3')	Repeat Type	T_m_	k	Ho	He	PIC
D15	D15F: 5' TGTGACCTTTCTCTAGTTTC D15R: 5' GCACAAAACATTCAGTCTCC	(CA)_22_, simple	51	8	0.381	0.740	0.689
3E6	3E6F: 5' CCTGGGGATAATAAAACTAGTA 2E6R: 5'CATGAATGAATCTTTACACTGA	(TAA)_11_, simple	56	5	0.571	0.684	0.606
E21B	E21F: 5' GCGATAAAGGCTGGCAGAGG E21BR: 5' CTTTGAGGGTCTGTTCTACTGTGA	(CA)_21_, simple	61	5	0.667	0.718	0.657
D10	D10F: 5' CCCTCTCTGTCCCTCCCTTG D10R: 5' GCCGTTTCCCTCATGCTACA	(GT)_14_, simple	63	5	0.524	0.812	0.759
E7	E7F: 5'GCCCCAAAGCCCTAAAATAA E7R: 5'GCATGTCGGACAGTAAAGCA	(CA)_11_CG(CA)_4_, interrupted	55	7	0.571	0.662	0.600
C34	C34F: 5'CTCCACACTGAGCATGGAAA C34R: 5'CAACCAAAGGCAGGAACAGT	(CT)_21_, simple	55	8	0.250	0.805	0.756
6HDZ003	Williamson *et al *(2002)		55	8	0.350	0.782	0.729
Fca304	Menotti-Raymond *et al*, 1999, Luo S-J *et al*, 2004		55	8	0.476	0.753	0.701
Fca43	Menotti-Raymond *et al*, 1999, Luo S-J *et al*, 2004		55	6	0.524	0.769	0.714
Fca139	Menotti-Raymond *et al*, 1999, Luo S-J *et al*, 2004		55	4	0.167	0.678	0.600

Tm = Annealing temperature; k = number of alleles; He = Expected heterozygosity; Ho = Observed Heterozygosity; PIC = Polymorphism Information Content

**Figure 4:**
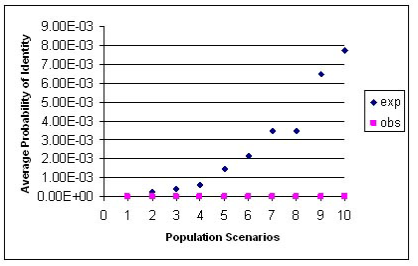
**Plot of the observed and expected P(ID) values of six loci on different population scenarios with different proportions of related individuals**. The greater the number of siblings in the population, the higher is the average probability of identity and therefore the higher will be the number of loci required to distinguish individuals. The relationship proportions are numbered as: **1 **= all unrelated animals; **2 **= 0.1 siblings, 0.2 parents, 0.5 half siblings; **3 **= 0.2 siblings, 0.2 unrelated, 0.2 parents, 0.4 half-siblings, 0.2 unrelated; **4 **= 0.3 siblings, 0.2 parents, 0.3 half siblings, 0.2 unrelated; **5 **= 0.5 siblings, 0.2 parents, 0.1 half-siblings, 0.2 unrelated; **6 **= 0.6 siblings, 0.2 parents, 0 half-siblings, 0.2 unrelated; **7 **= 0.7 siblings, 0.2 parents, 0 half-siblings, 0.1 unrelated; **8 **= 0.8 siblings, 0.2 parents, 0 siblings, 0 unrelated; **9 **= 0.9 siblings, 0.1 parents, 0 unrelated; **10 **= 1.0 siblings.

**Figure 5:**
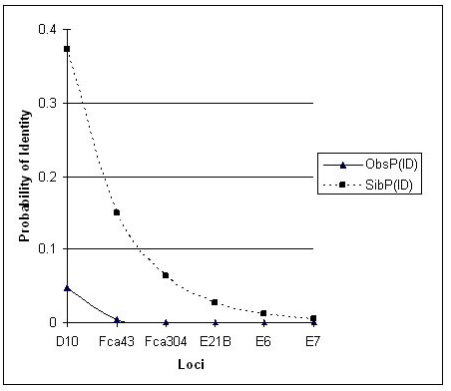
**Decrease in the P(ID) values with the addition of loci in decreasing order of heterozygosity**. Observed and Sibling P(ID)s were calculated for six microsatellite loci on 21 captive tigers. Cumulative Observed Probability of Identity becomes zero with the three most informative loci.

Loci E21, E6, D10, Fca43, E7 and Fca304 were used for genotyping as they amplify fragments lesser than 200 bp and have expected heterozygosity of 0.6 or more. Plot of different relationship scenarios with the P(ID) values shows that the value increases for a population scenario that comprises of high proportion of siblings (Figure 4). Plot of observed and sibling P(ID) values show that the six microsatellite markers have a sibling P(ID) of 0.005, i.e., these markers can distinguish with 99.9 % probability even siblings (Figure 5).

### Genotyping of faecal samples

The captive tiger reference genotypes and the faecal genotypes are compared manually (Table 2). There is one case of spurious amplification at locus E7 (Roger/Scat1), which is a 3.3% error rate for all the consensus genotypes generated.

**Table 2:** Comparison of reference genotypes generated from tiger blood samples with genotypes generated from tiger scat samples.

Individual	Sample type	E21B	E6	E7	D10	Fca304
Roger	Blood	162/158	153/153	142/142	145/134	137/121

	Scat1	162/158	153/153	142/**136**	145/134	137/121
	Scat2	162/158	153/153	142/142	145/134	137/121
	Scat3	162/158	153/153	142/142	145/134	137/121

Tina	Blood	160/160	153/153	140/140	144/144	137/137

	Scat1	160/160	153/153	140/140	144/144	137/137
	Scat2	160/160	153/153	140/140	144/144	137/137
	Scat3	160/160	153/153	140/140	144/144	137/137

One spurious allele noted at Roger/Locus E7/Scat 1.

Of the 48 samples that were collected from Mudumalai and BRT Wildlife Sanctuaries, DNA could be extracted from 40 samples (83%) as was confirmed by amplification with 146 bp cytochrome b primers [29]. Of these, 28 samples (70%) were positive for tiger as noted by the presence of amplicons with the mitochondrial tiger-specific PCR. Of the samples that were positive for tiger, microsatellite DNA could be amplified from 17 samples (60%), which include partial as well as complete profiles. Complete genotype profiles could be obtained for 10 samples (58%) and partial profiles for 7 samples (41.1%). When partial profiles were obtained, they were matched and grouped with the complete profiles only if they matched at least at four of the most informative loci namely D10, Fca43, Fca304 and E21B (in that order). Partial profiles that did not match at these loci with complete profiles were excluded from the analysis. The subsequent analysis was performed only with those loci for which consensus genotypes were obtained.

The total number of positive PCRs to obtain the 93 consensus genotypes of Mudumalai and BRT Sanctuaries, using the Comparative Multiple Tubes (CMT) approach, for both complete as well as partial profiles was 378, i.e., an average of 4 PCR reactions. Analysis of the genotypes obtained from Mudumalai Wildlife Sanctuary (Table 3) showed that profiles of samples M3 and M6 completely match each other. Sample M7, though a partial profile did not match at any of the three most informative loci namely D10, Fca43 and Fca304 with the consensus genotypes of the rest of the genotypes and was therefore considered as a new genotype. Ten samples had to be excluded from the grouping, as all the three informative loci did not amplify. Amongst the BRT Wildlife Sanctuary samples, partial profiles of samples BN11 and BN12 were grouped together as were the partial profiles of samples BN14 and BN15 on the basis of the three most informative loci. Among the Mudumalai and BRT genotypes we identified 13 unique profiles (Mudumalai = 6; BRT = 7) that are presumably from 13 individual tigers.

**Table 3:** Consensus genotypes and sex identification for the faecal samples obtained from Mudumalai Wildlife and BRT Wildlife Sanctuaries.

Mudumalai Wildlife Sanctuary							
Sample	E21B	Fca43	E6	E7	D10	Fca304	Zn and Aml
M3	158/158	117/111	153/147	148/148	H/140	131/139	Female
M6	158/158	117/111	153/147	148/148	140/140	131/139	Female

M7	152/H	117117	--------	148/142	144/134	135/135	Female

M10	158/166	117/111	147/147	142/140	144/144	H/131	Male

M11	158/158	111/H	159/147	142/142	144/140	135/129	Female

M27	158/166	117/111	159/153	148/142	144/140	H/129	Female
M25	158/158	117/111	158/153	148/142	144/140	129/135	Female

M32	156/166	117/111	---------	142/140	146/142	129/135	NA

BRT Wildldife Sanctuary							

BN1	158/158	115/115	147/153	148/148	H/140	131/139	Female

BN4	158/158	119/115	147/147	142/148	142/144	125/139	Female

BN6	158/158	---------	163/163	150/152	136/136	117/117	Male

BN7	158/158	117/115	147/147	148/148	134/140	139/139	Female

BN11	158/H	115/115	---------	142/148	134/144	----------	NA
BN12	158/158	---------	156/156	142/148	134/144	125/129	NA

BN14	158/158	---------	155/155	H/146	140/144	131/133	NA
BN15	158/158	---------	155/155	142/H	140/144	----------	Male

BN3A	156/156	117/117	147/H	142/148	144/144	135/135	Female

Zn and AML are the zinc-finger and amelogenin PCRs that were used for sex identification of the tiger scat samples. 'H' indicates that the locus was scored as a half-locus because one additional allele was scored in one of the five amplifications to obtain consensus. Dashed lines indicate that enough PCRs could not be amplified for obtaining consensus genotypes. NA indicates that PCR amplification could not be obtained.

Dropout rate in faecal genotypes generated from the field-collected samples was considerably higher than that from the zoological park samples (Table 4). All the faecal samples collected from the captive tigers amplified (100%). Captive tiger DNA samples of known sex were PCR-amplified with the zinc-finger and amelogenein primers [30]. With the zinc-finger PCR two amplicons of 164 and 161 bp were obtained for male tigers and one amplicon of 164 bp was obtained for females. Similarly, for the amelogenin PCR, two amplicons of 216 and 194 bp were obtained in male tigers as against one amplicon of 216 bp for females.

**Table 4:** Comparison of the rate of allelic dropout and appearance of false alleles in captive tiger scat samples and tiger scat samples collected from the field.

	**Captive tiger samples**		**Field-collected samples**	
Locus	Dropout	False allele	Dropout	False allele

E21B	0.067	0.000	0.394	0.020
E6	0.333	0.025	0.250	0.013
E7	0.000	0.038	0.466	0.000
D10	0.000	0.000	0.501	0.090
Fca304	0.000	0.000	0.648	0.000
Fca43	0.000	0.000	0.000	0.018

Mudumalai faecal samples number M10 and BRT Sanctuary sample BN6 and BN15 gave two PCR products with both the zinc-finger and the amelogenin PCR with sizes corresponding to male tiger. Non-specific bands of sizes corresponding to 202 base pairs were obtained in samples BN4, BN6 and M3, which may have been obtained from prey DNA that would have co-purified with faecal DNA. Out of the 17 genotypes, partial as well as complete, sex identification reaction did not work on 4 samples (23.5%) i.e., on samples BN11, BN12, BN14 and M32.

## Discussion

Traditionally, faecal matter of tigers has been used to study the food habits of tigers and their sympatric carnivores. As they are more readily obtainable than hair, faecal samples were chosen as the non-invasive source for obtaining DNA for our study. DNA quantity and quality by different extraction trials was evaluated. The DNA extracted from faecal samples originates from the colorectal epithelial mucous on the surface of the sample. Also it appears that the DNA that is extracted from the surface of the sample has less of PCR inhibitory substances and possibly less admixture with undigested prey remains [31]. Thus, in all the five DNA extraction methods that were evaluated here, the surface of the faecal sample was washed with the initial Lysis buffers. Three methods, namely the Digest Buffer/Phenol-Chloroform method, Guanidinium thiocyanate-silica method and Qiagen Stool DNA extraction kit showed PCR amplification with no significant difference. Guanidinium thiocyante-silica was followed for all our extractions.

We used PCR amplification to evaluate the best methods for storing the faecal samples after collection. Both the storage methods tested, namely alcohol as well as silica beads, have worked well in terms of PCR amplification ability and can be used to preserve faecal samples of carnivores. This was not tested exclusively in the faecal samples of tiger as we collected samples of tiger as well as its sympatric carnivores, leopard and wild dog. The faecal samples collected at NSTR were also used in scat identification trials discussed next.

It is essential to reliably identify the faecal samples of tiger from those of their sympatric carnivores like leopards and wild dog. Most studies on diets and occupancy of the two felids, namely the tiger and the leopard, are based on faecal samples [32,33], which are identified on the basis of morphological features [34] and associated behavioral signs like scrape marks, the absence of which makes it difficult to identify the samples. Therefore, molecular analysis that makes use of tiger-specific mitochondrial variations for distinguishing the tiger faecal samples from those of sympatric carnivores was developed and used to screen out tiger samples.

According to Davison *et al *[35], sometimes even trained trackers have misidentified the faecal samples of similar sized carnivores. For instance, in our field study at the Mudumalai and BRT Wildlife Sanctuaries, only 70% of all the samples that were collected as supposed tiger faecal samples were actually of tiger origin. False negatives may occur in the tiger-specific PCR generated as a result of interspecific polymorphisms at the variable sites that were used in primer design. We ruled this out by PCR amplifying DNA of 25 tigers and 10 leopards DNA (data not shown) and also by amplifying representative samples of some of the animals that are the prey of tigers in India.

Microsatellite Loci E21B, E7, E6, Fca43, D10 and Fca304 target fragments of less than 200 bp long and are therefore easier to amplify [36]. Hence they were chosen for our study. The most conservative estimate of P(ID) i.e., P(ID)_sib _is determined to estimate the upper bound on the number of loci required to reliably distinguish even closely related individuals. The set of primers used in this study are sufficient to distinguish a population comprising even of several siblings (Figure 4). For individual identification in population estimation studies using mark-recapture models, P(ID) value is recommended to be approximately 0.01 [19] in order to distinguish closely related individuals with 99% certainty. Using microsatellite loci with high heterozygosity reduces the number of loci required to reach a low P(ID) value. The P(ID)_sibs _for the six loci used by us is 0.005 and can be used to distinguish even closely related individuals. Therefore they can also be used in studies requiring individual identification for population estimation. Further, these loci are in Hardy-Weinberg equilibrium and not in linkage disequilibrium – an essential assumption required in calculating P(ID) where loci are expected to be independent. In our study, the observed P(ID) for the three most informative loci is zero. The P(ID) value calculated in our study was with captive tigers which may be inbred. It is therefore possible that the number of loci required to distinguish individuals can stand valid even in those wild tiger populations that may comprise of many related tigers.

The Comparative Multiple Tubes [22] approach was used to derive consensus genotypes for faecal DNA extracts. Reference genotype profiles were generated from blood DNA of known captive tigers that were compared with genotypes generated from faecal samples of the same animals (Table 2). Though there is one erroneous genotype, which might be an amplification artifact at one locus ('Roger', Locus E7, Scat1), the profiles generated from the captive samples were assigned with the corresponding reference profiles on the basis of three of the most informative loci, namely D10, Fca304 and E21B.

In order to evaluate whether faecal samples collected from the jungles could be used for genotyping and to demonstrate the error rates in the genotypes generated from such samples, a preliminary study was performed on faecal samples collected from Mudumalai and BRT Wildlife Sanctuaries. It is generally believed that the length of time between deposition of samples and collection and extraction may have some influence on the DNA quantity [37]. This is evident as the faecal samples collected from captive tigers were amplified (microsatellite loci) with 100% success as against the 60% amplification success with faecal samples from the field. Also the rate of errors generated from captive animal faecal samples is considerably lower than the errors generated from faecal samples collected from the jungle. This could be because the DNA from faecal samples of the captive animals was extracted on the day of collection. The error rate encountered by us for faecal samples collected from the field was in the same range as has been reported by others, i.e., 46–66% [20,23], though others have reported a much higher amplification success rate – 87% [22] and 93–95% [38].

As faecal samples were collected wherever encountered from all over the two protected areas, the number of genotype profiles generated thus can provide an estimate of the minimum number of tigers living in that area during that particular sampling session. Partial genotype profiles were grouped together with those samples where complete genotypes could be obtained. This kind of assignment could lead to an underestimation, but not an overestimation of animal numbers.

Sex identification of faecal samples can reveal the sex ratio of tigers, as well as territory occupancy by male and female tigers in a habitat. PCR primers that have been developed for several New World felids [30] and which target the zinc-finger region and amelogenein gene on the X and Y-chromosome were used for sexing faecal samples. We tested these primers on known captive female and male tiger DNA samples extracted from blood before using them on faecal DNA extracts.

This pilot study demonstrates the use of faecal samples of tigers collected in the field as a source of DNA and the possibility of conducting population estimation wherein matching genotypes are considered to arise from the same individual. The genotype data generated thus could be analysed by mark-recapture analysis [39] with appropriate models.

## Conclusion

The results of this study demonstrate the feasibility of using non-invasive genetics as one of the methods for monitoring as well as estimating tiger populations in protected areas. However it is our contention that population studies of a cryptic species like tiger should be carried out using more than one method, with the genetics component playing a predominant role, in monitoring and estimation of tiger populations for conservation planning and management.

## Methods

### Sample collection

For DNA extraction trials, fresh faecal samples (n = 10) were collected from captive tigers, housed at the Nehru Zoological Park, Hyderabad, India. For faecal sample preservation and wild tiger faecal sample identification trials, carnivore scats (n = 8) were collected from the NSTR, Andhra Pradesh during the Tiger Census conducted by the Andhra Pradesh Forest Department in January 2001. Each sample was divided into two portions and preserved separately in 90% ethanol and silica gel pouches contained in 50 ml screw-cap tubes and kept at room temperature (Average air temperatures were 37°–40°C at the time of collection) and transported to the laboratory where DNA was extracted within a week of receiving the samples. DNA samples of different animals for validation of the tiger-specific PCR primers were obtained from the 'DNA Bank' at the Centre for Cellular and Molecular Biology (CCMB), India.

DNA extracted from blood samples of captive tigers (n = 21) from different zoological parks of India (Bhubaneshwar Zoological Park, Orissa; Nehru Zoological Park, Hyderabad, Alipore Zoological Park, Calcutta; Bannerghatta Biological Park, Bangalore; Shimoga Zoo, Shimoga) available at the 'DNA Bank' of CCMB were used for polymorphism study and calculations of P(ID).

To test for genotype reliability, faecal samples (n = 3) were collected from two tigers, namely 'Tina' (female) and 'Roger' (male) for which blood DNA was available.

In order to assess actual genotyping error rates for the field study, faecal samples were collected from Mudumalai Wildlife Sanctuary (n = 34) Tamil Nadu and Biligiri Rangan Temple (BRT) Wildlife Sanctuary (n = 15) in May 2003 and May 2005, respectively. Samples were collected with the help of trackers of the local Forest Department along animal trails and tracks. GPS locations of the samples were taken and plotted on toposheets (not shown).

### Faecal sample preservation

DNA from carnivore faecal samples of NSTR stored in storage desiccant silica or 90% ethanol was extracted by the Guanidinium-thiocyanate method, PCR-amplified with cyotchrome b primers. Positive PCRs were scored as 1 and non-amplification was scored as 0. The difference between the two preservation methods was checked with two-tailed t-test in Excel.

### DNA extraction

DNA was extracted from the faecal samples collected from captive tigers by the following five methods: The Chelex-100 method [25]; the Digest Buffer/Phenol Chloroform method [26,27]; the Lysis buffer/column purification method [28]; Guanidinium thiocyanate-silica method as modified by Reed *et al *[26] and Qiagen Stool DNA extraction kit. PCR amplification of all the extracts was carried out first with mitochondrial cytochrome b PCR primers, following which extracts from buffer/column purification, Guanidinium thiocyanate-silica and Qiagen Stool DNA extraction kit methods were amplified with the microsatellite markers developed and described later. The presence of bands was scored as 1 and the absence as 0. Non-parametric Tukey-type multiple comparison (Nemenyi test) was performed to look for difference between the three extraction methods.

### Molecular identification of faecal samples

A PCR-based assay was developed to reliably identify the faecal samples of tigers from those of other sympatric carnivores, especially leopards. Mitochondrial cytochrome b sequences of the Bengal tiger,*Panthera tigris tigris *[GenBank: AF053019–AF053025], its sympatric carnivore leopard, *Panthera pardus *[GenBank: AY005809] and some of the animals preyed upon by tiger namely sambhar *Cervus unicolor *[GenBank: AF423201], barking deer *Muntiacus muntjac *[GenBank: AY225986], wild pig *Sus scrofa *[GenBank: AY237529], hog deer *Axis porcinus *[GenBank: AY035874], Indian bison *Bos gaurus *[GenBank: AF348593], spotted deer *Cervus axis *[GenBank: AY182236], domestic goat *Capra hircus *[GenBank: AB110595], domestic buffalo *Bubalis bubalus *[GenBank: D82894] were downloaded. All the sequences were aligned in ClustalX 1.8 with *Panthera tigris tigris *[GenBank: AF 053018] as the reference sequence, to identify nucleotides that were found exclusively in tiger but not in the other species. ARMS (Amplification Refractory Mutation System) PCR primers were designed such that the 3' ends of the forward and reverse primers were on bases specifically found in tiger and not in sympatric carnivores or prey animals. The primer sequences are:

TIF: 5'-ATAAAAAATCAGGAATGGTG-3'

TIR: 5'-TGGCGGGGATGTAGTTATCA-3'

Initially these primers were tested by *in silico *PCR on GenBank sequences with the Amplify 1.2 software (Engels B, Department of Genetics, University of Wisconsin, Madison WI 5706) following which they were PCR amplified with DNA samples of some animals namely, Bengal tiger (*Panthera tigris tigris*), Siberian tiger (*P.t.altica*), leopard (*P. pardus*), lion (*P. leo*), clouded leopard (*Neofelis nebulosa*), domestic dog (*Canis familiaris*), wild dog (*Cuon alpinus*), wolf (*Canis lupus*), jackal (*Canis aureus*), goat (*Capra hircus*), wild pig (*Sus scrofa*), Indian bison (*Bos gaurus*), Spotted Deer (*Cervus axis*), Sambar (*C. unicolor*) and human (*Homo sapiens*).

For identifying the faecal samples collected from the wild, DNA extracted from samples collected from the NSTR were PCR-amplified with TIF/TIR primers. Positive amplicons were sequenced on an ABI 3730 Automated DNA Sequencer. Sequences obtained were aligned using ClustalX 1.2 with GenBank sequences of a reference tiger (AF053019), some prey animal species of tiger and leopard, the sympatric felid. GENEDOC software was used for formatting the sequences. A phylogeny analysis of aligned sequences was done with MEGA v3.1 software [40]. NJ tree was constructed with the sequences using Interior Branch Test of Phylogeny with default values for the number of replicates.

### Development of polymorphic microsatellite markers

A small insert partial genomic library was developed in pMOS*blue *Blunt-ended cloning vector from DNA of a captive tiger housed at the Nehru Zoological Park, Hyderabad, by methods described elsewhere [41,42]. Twenty-three microsatellite repeat containing clones were sequenced on an ABI 3700 Automated DNA sequencer and 15 microsatellite-containing clones were used to design primers from the region flanking the microsatellite repeat using the software Genetool. Forward primers (BioServe Biotechnologies, India) were labelled at their 5'-end with FAM or HEX fluorescent label. Microsatellite primers were screened for polymorphism on DNA samples of captive tigers (n = 21). Microsatellite primers 6HDZ003 and 6HDZ007 developed for Sumatran tiger [43] and Domestic cat primers Fca45, Fca90 [44], Fca212, Fca310, Fca304, Fca43 and Fca139 [45], were also screened to look for polymorphic markers.

The software CERVUS was used to calculate Polymorphism Information Content (PIC). ARLEQUIN software was used to calculate expected (H_e_), observed heterozygosity (H_o_) and deviations from Hardy-Weinberg equilibrium, as well as, linkage disequilibrium between the loci. Bonferroni correction was applied to correct for Type II errors.

### Probability of Identity, P(ID)

Probability of Identity, P(ID), was calculated for six loci namely E7, E21B, E6, D10, Fca304 and Fca43. A population may comprise of closely related individuals other than just siblings, like half-siblings or parent-offspring, etc. Different relationship scenarios were included in the calculation of the expected and observed P(ID) values with API-CALC software [46]. Observed P(ID) was calculated with the software API-CALC and siblings P(ID) with software GIMLET v1.3.3 [47] for the captive tigers genotype data (n = 21). The Observed and the Sibling P(ID) of the locus with the highest heterozygosity was taken first and the cumulative probability of the rest of the loci calculated as the product of the probabilities of the next most heterozygous locus and so on.

### PCR amplifications

For identifying the best method of DNA extraction and comparing the two preservation methods, PCR amplifications were carried out with mitochondrial cytochrome b primers targeting a 146 bp fragment of DNA [29].

For identifying tiger faecal samples PCR amplifications with tiger-specific cytochrome b primers TIF/TIR was carried out in 25 μl reactions with the following final concentration: 1× PCR Buffer II (Applied Biosystems), 1.5 mM MgCl_2 _(Applied Biosystems), 0.15 mM dNTPs, 1× BSA (New England Biolabs), 1U Ampli*Taq*Gold, 5 μl each of primers TIF and TIR and 3 μl template DNA. PCR reactions were carried out in MJ Research PTC-200 Thermal Cycler with the following conditions: 95°C for 10 mins, 40 cycles of 95°C for 45 secs, 59°C for 30 secs (Ramp: 0.5 Deg/Sec), 72°C for 30 secs, followed by a final extension of 72°C for 10 mins.

All microsatellite PCR reactions were carried out in 15 μl reactions: 1XPCR Buffer II (Applied Biosystems), 1.5 mM MgCl_2 _(Applied Biosystem), 1× BSA (New England Biolabs), 0.15 mM dNTP (Applied Biosystems), 1 Unit of Ampli*Taq*Gold DNA polymerase (Applied Biosystems), 5 picomoles of the forward and reverse primers each and 10 ng of template DNA. T_m _values of the primers are listed in Table 1.

All genotyping reactions were carried out on an ABI 3730 Automated DNA sequencer with ROX-500 size standard (Applied Biosystems) and analyzed on Genemapper v3.7 software (Applied Biosystem).

### Genotyping faecal samples

For genotyping DNA from faecal samples, the Comparative Multiple Tubes (CMT) approach [22] was used. For testing reliability of genotypes, reference genotypes generated at loci E21B, E6, E7, D10 and Fca304 from blood DNA of two captive tigers were compared to genotypes from faecal DNA extracts from both the animals.

DNA was extracted from all the faecal samples collected from Mudumalai and BRT Wildlife Sanctuaries. All extracts were tested for the presence of good quality DNA with cytochrome b primers [29] followed by PCR amplification with tiger-specific cytochrome b primers. Tiger positive samples were analyzed further with the set of six microsatellite primers. Samples for which complete profiles could not be obtained for all the loci were grouped along with those samples for which complete genotypes could be obtained on the basis of consensus genotype match at least at three of the four most heterozygous loci namely, D10, Fca43, Fca304 and E21B (in that order). Consensus fingerprints were obtained by pairwise comparison of the genotypes [22]. Similar genotypes were grouped together manually. Rates of allelic dropouts and false amplifications from captive tiger samples and field-collected samples were computed with GIMLET 1.3.3 software [47] and compared. Mudumalai and BRT Sanctuary faecal samples were PCR-amplified in duplicate with FAM-labeled forward primers targeting zinc-finger region and the amelogenin gene for sex identification, and products were sized on an ABI 3730 Automated DNA sequencer and analyzed with the Genemapper software.

## Authors' contributions

JB carried out the sample collection, molecular genetic studies, statistical analysis, participated in the design of the study and wrote the manuscript. LS conceived of this study and its design, edited the manuscript and supervised JB. Both the authors read and approved the final version of the manuscript.
